# Zebrafish *snai2* mutants fail to phenocopy morphant phenotypes

**DOI:** 10.1371/journal.pone.0202747

**Published:** 2018-09-12

**Authors:** Cara Bickers, Sophia D. Española, Stephanie Grainger, Claire Pouget, David Traver

**Affiliations:** Department of Cellular and Molecular Medicine and Section of Cell and Developmental Biology, University of California, San Diego, La Jolla, CA, United States of America; Rutgers-Robert Wood Johnson Medical School, UNITED STATES

## Abstract

Snail2 is a zinc-finger transcription factor best known to repress expression of genes encoding cell adherence proteins to facilitate induction of the epithelial-to-mesenchymal transition. While this role has been best documented in the developmental migration of the neural crest and mesoderm, here we expand on previously reported preliminary findings that morpholino knock-down of *snai2* impairs the generation of hematopoietic stem cells (HSCs) during zebrafish development. We demonstrate that *snai2* morphants fail to initiate HSC specification and show defects in the somitic niche of migrating HSC precursors. These defects include a reduction in sclerotome markers as well as in the Notch ligands *dlc* and *dld*, which are known to be essential components of HSC specification. Accordingly, enforced expression of the Notch1-intracellular domain was capable of rescuing HSC specification in *snai2* morphants. To parallel our approach, we obtained two mutant alleles of *snai2*. In contrast to the morphants, homozygous mutant embryos displayed no defects in HSC specification or in sclerotome development, and mutant fish survive into adulthood. However, when these homozygous mutants were injected with *snai2* morpholino, HSCs were improperly specified. In summary, our morpholino data support a role for Snai2 in HSC development, whereas our mutant data suggest that Snai2 is dispensable for this process. Together, these findings further support the need for careful consideration of both morpholino and mutant phenotypes in studies of gene function.

## Introduction

The small pool of hematopoietic stem cells (HSCs) derived during embryogenesis gives rise to the lifelong supply of all blood cells. HSCs are derived from the ventral wall of the dorsal aorta through a process referred to as the endothelial-to-hematopoietic transition (EHT)[1–6]. Before this event can occur, however, HSC precursors are directed by a complex cascade of signaling events. During development, both hematopoietic and endothelial precursors, which are derived from posterior lateral mesoderm (PLM), migrate beneath the somites to the embryonic midline to form the trunk vasculature[7,8]. During this migration, the somites provide instructional cues, including canonical Wnt[9] and Notch signaling[7,10,11], which culminate in specifying the cellular identity of hemogenic endothelium. Upstream of these signals, there is a complex network of other intrasomitic signaling events, including non-canonical Wnt and FGF signaling[10,12]. In addition, specific compartments of the somites, including the sclerotome[10,11] and endotome[13], have also been identified as essential players in embryonic hematopoiesis. This is likely due to the compartmental specific relays required during the complex signaling cascade that forms the developmental HSC niche, a process that is incompletely understood. Further understanding of the niche will contribute to a major goal in regenerative medicine: reproducing the proper supportive environment for HSC instruction *in vitro*[14].

Using a genomics based approach, we previously identified *snai2* as upregulated in stromal cell lines supportive of HSCs *in vitro*[15]. The Snail family of zinc-finger transcriptional repressors includes three members: *snai1/snail*, *snai2/slug*, and *snai3/smuc*[16,17]. These repressors play critical roles during the epithelial-to-mesenchymal transition (EMT), and have been best studied in processes including gastrulation, neural crest delamination, and heart valve formation[18,19]. Snail2 has been shown to cell-intrinsically protect adult hematopoietic progenitor cells from radiation induced apoptosis[20], likely due to blocking self-renewal and proliferation[21]. *Snai2*^*-/-*^ mice develop macrocytic anemia and impaired T-cell maturation; however, no defects in HSC specification, emergence, or differentiation have been reported[22]. In addition, redundancy of the Snail family in the hematopoietic system has been implicated, since the combined loss of Snai2 and Snai3, but not loss of either gene alone, leads to fatal lymphoid dysfunction[23,24].

To investigate *snai2* function in zebrafish, we used morpholino knock-down and mutant zebrafish alleles, where we identified inconsistencies between mutants predicted to have Snail2 loss of function and animals subjected to morpholino knock down—a phenomenon also observed by groups targeting other genes[25–29]. Our knock down studies support a requirement for Snai2 in HSC development, upstream of Notch signaling, likely by promoting sclerotome formation. However, our mutant studies suggest that Snai2 is dispensable during HSC development, and that the Snai2 morpholino may have off-target or toxic effects. Overall, the discrepancies we observed between morpholinos and genetic mutants support the need to review and revise use guidelines for both morpholinos and mutants, as has been suggested by other groups[28,30].

## Materials and methods

### Zebrafish husbandry

Zebrafish were maintained and propagated as previously described[31], and this study was approved by the University of California at San Diego Institutional Animal Care and Use Committee (UCSD IACUC) under protocol number S04168. Embryos and adult fish were raised in a circulating aquarium system (Aquaneering) at 28°C. All anesthesia was completed with 100 mg/L Tricaine solution, and euthanasia of adult fish was completed with an ice slurry. The following zebrafish strains were used: wildtype AB*, *Tg(CD41*:*GFP)*[32], *Tg(kdrl*:*mCherry)*[33], *Tg(Gata2b*:*Gal4/UAS*:*LA-GFP)*[34], *Tg(actc1b*:*GFP)*[35], *Tg(TP1*:*GFP)*^*um14*^ [36], *Tg(5xUAE-E1b*:*6xMYC-notch1a)*^*kca3*^ [37] referred to as *Tg(UAS*:*NICD-myc)* for simplification, *Tg(kdrl*:*miniGal4)*[11], *snai2*^*sa24539*^ [38], *snai2*^*112Δ*^, and *p53*^*-/-*^ [39]. The deletion mutant of *snai2* was produced utilizing the CRISPR/Cas9 genomic editing system. gRNAs were designed utilizing the CHOP-CHOP web-tool and coninjected with Cas9 mRNA, as previously described [40–42]. gRNAs were designed to target three sequences within the *snai2* gene: CCTCAGCCTGAAGTGTTAAGCCC, CCCCTTCCCCACGACCTGTCCCC, and CCTCATCTCTCTCTGACACATCC. A 112 base pair deletion, predicted to result in frame-shift and early termination was isolated and propagated for studies. The allele number of this mutant is #SD57.

### Whole mount *in situ* hybridization (WISH) and histology

WISH and double fluorescent WISH were conducted as previously described[12,43], using established DIG-labelled or fluorescein labelled anti-sense RNA probes including *runx1*, *foxc1b*, *dlc*, *dld*, *cmyb*, *gata1*, *fli1a*, *kdrl*, *dll4*, *cdh17*, *rag1*, and *jam2*[7,10]. Antisense probes for *snai2*, *snai1a*, *snai1b*, and *pax9* were designed to target the entire coding region. Following WISH, selected embryos were processed for cryosectioning, according to standard procedures on the Leica CM1860 Cryostat at 10 μm thickness. WISH and Cryosections were imaged on a DFC295 digital camera using the Leica FireCam Software. All confocal images of double fluorescent *in situ* hybridization and fluorescent transgenic embryos were obtained with a Leica SP5 inverted confocal microscope (Leica Microsystems).

### Microinjections of mRNA and morpholino

Embryos were injected at the one-cell stage with morpholino oligonucleotides (MOs, GeneTools) and/or mRNA. Antisense MOs were used at the following concentrations: 10 ng *snai2* splice-block MO (SB MO)[15,44] and 0.75 ng *snai2* 5’UTR MO (UTR MO). Capped mRNA was synthesized from linearized pCS2^+^ constructs using the mMessage mMachine SP6 Transcription Kit (ambion, AM1340), according to manufacturer’s recommendations. Full length *snai2* mRNA was injected into embryos at 150 ng/μl.

### Cell Sorting and real-time quantitative PCR (qPCR)

Double transgenic *Tg(CD41*:*GFP/kdrl*:*mCherry)* or *Tg(actc1b*:*GFP)* embryos were prepared for flow cytometry at 48 hpf and 17 hpf, respectively as previously described[45]. Single, live cells were sorted on a FACSAria II and collected for RNA extraction. Total RNA was extracted from pools of embryos via TRIzol (Ambion) and sorted cells using the RNEasy Mini Kit (Qiagen, 74104), cDNA was synthesized using iScript gDNA Clear cDNA Synthesis Kit (Bio-Rad), and RT-qPCR reactions were performed using SYBR Green (Bio-Rad) and a Bio-Rad CFX96 real time system according to the manufacturer’s instructions. The expression of housekeeping genes *ef1α* and *β-Actin* were used to normalize using the ΔΔC_t_ method. Primer sequences are listed in Table 1.

**Table 1:** qPCR primers.

Gene	Forward	Reverse
***ßactin***	cgtctggatctagctggtcgtga	caatttctctttcggctgtggtg
***cmyb***	agagggtgaaagaaatcgag	actgaaacaacaatgccaac
***dlc***	acgagcagtgtgtgtaa	tgttattctctgttgactgg
***dld***	agtactgcacagaacc	tcttggttacagaagag
***ef1a***	gagaagttcgagaaggaagc	cgtagtatttgctggtctcg
***kdrl***	ctcctgtacagcaaggaatg	atctttgggcaccttatagc
***meox***	ggcttttaccaaagagcaac	tttgaaaccacactttcacc
snai1a	tgaagatgcacatccgctc	gttacagtgtgggcaggag
***snai1b***	tatccctccatgcttgtgtg	tcatcctcctctccactgct
***snai2***	accgaattatagtgaactggaga	actgttatgggattgtacgcc
***snai3***	cacacaggtgagaaaccatt	ttgatctcagagtgggtttg
***twist1b***	aatcatccccactttaccttc	gtgagcaacataactacaactt

### Immunohistochemistry (IHC)

NICD expression was confirmed using immunohistochemistry was completed with anti-c-myc antibody (1:1000, BioLegend) post-WISH, as previously reported[46]. Embryos were imaged for GFP fluorescence on the AxioZoom.V16 (Zeiss), followed by brightfield imaging on the Leica MZ16.

### *Snai2*^*112Δ*^ and *Snai2*^*sa24539*^ genotyping

*Snai2*^*112Δ*^ were generated as described above and genotyped using the following primers: forward 5’-ATGTGACCTGTCAAAGTATGGC-3’ and reverse 5’- TACACAAACCGCACTGAAACTT-3’. Zebrafish embryos with a nonsense point mutation in the second exon of *snai2* were obtained from the Zebrafish International Resource Center (ZIRC)[38]. The single nucleotide polymorphism (SNP) specific genotyping primers, designed as previously described[47], used are as follows: forward 5’- CACATCCTCTAATAAGGACCACAGCGGT-3’ and reverse 5’- GCTTCATGAGTCCCGAATACGTGTTG-3’.

### Whole kidney marrow analysis

Zebrafish kidneys were prepared for flow cytometry as previously described[48] and resuspended in 1x PBS with 1% fetal bovine serum. Samples were analyzed on a BD LSR II Flow Cytometer to separate cellular fractions by size and granularity as previously described[49].

## Results

### *Snai2* is expressed in the embryonic hematopoietic niche

**Fig 1:**
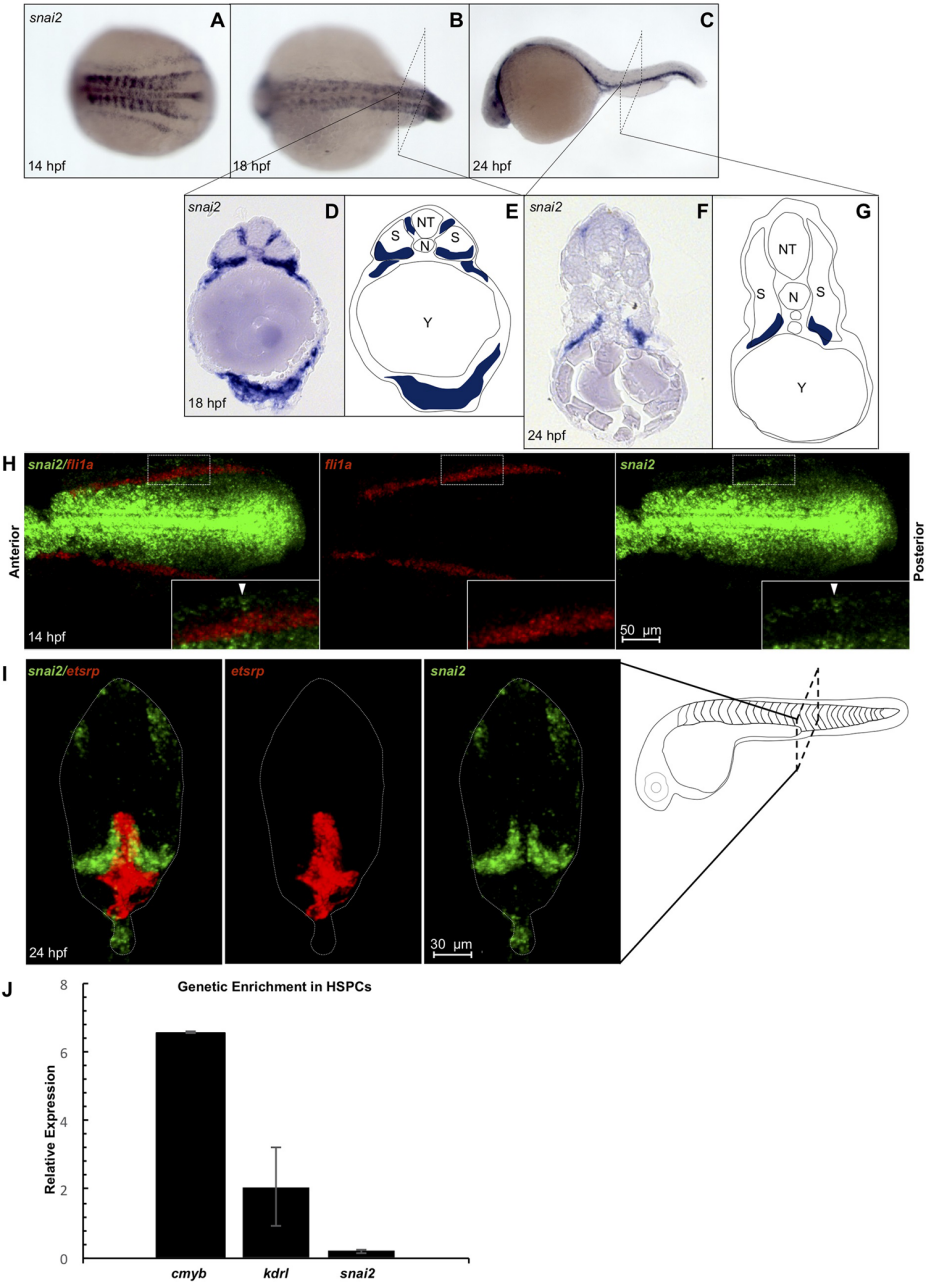
*Snai2* endogenous expression in wild-type embryos. Expression of *snai2* was analyzed via whole mount *in situ* hybridization at 14 hpf (A), 18 hpf (B), and 24 hpf (C). Embryos were cryosectioned post-*in situ* at 18 hpf (D) and 24 hpf (F). Simplified schematics are provided (E and G). Double fluorescent *in situ* for *snai2* with endothelial markers *fli1a* at 14 hpf (H) and *etsrp* at 26 hpf (I) was performed. Insets show a close-up view of the PLM. QPCR was used to compare *snai2* enrichment within double positive HSPCs sorted from *Tg(CD41*:*GFP/kdrl*:*mCherry)* on 2 dpf to the rest of the embryo. Markers *cmyb* and *kdrl*, which should be enriched in this population, are displayed alongside for comparison. N: notochord; NT: neural tube; S: somite.

To explore the role of Snail2 in embryonic hematopoiesis, its expression pattern was observed via whole-mount *in situ* hybridization (WISH) throughout early development. At 14 hours post fertilization (hpf), when the PLM has not yet begun its migration to the midline, *snai2* is highly expressed in the nervous system, somites, and an outer stripe of the mesoderm (Fig 1A). This expression pattern continues throughout somitogenesis, with somitic expression becoming localized to the most ventral portion of the somites (Fig 1B–1G), the tissue that most closely contacts migrating HSC precursors. At both 14 hpf (pre-migration) and 24 hpf (post-migration), *snai2* transcript showed no colocalization with the endothelial markers *fli1a* or *etsrp* via double fluorescent *in situ* hybridization (Fig 1H and 1I). QPCR from FACS purified hematopoietic stem and progenitor cells (HSPCs) and endothelial cells at 48 hpf demonstrated that *snai2* was not enriched in either population, as compared to all other cells in the embryo (Figs 1I and S1), suggesting that Snai2 function is not required cell-intrinsically.

### *Snai2* Morpholinos lead to a depletion of HSPCs

**Fig 2:**
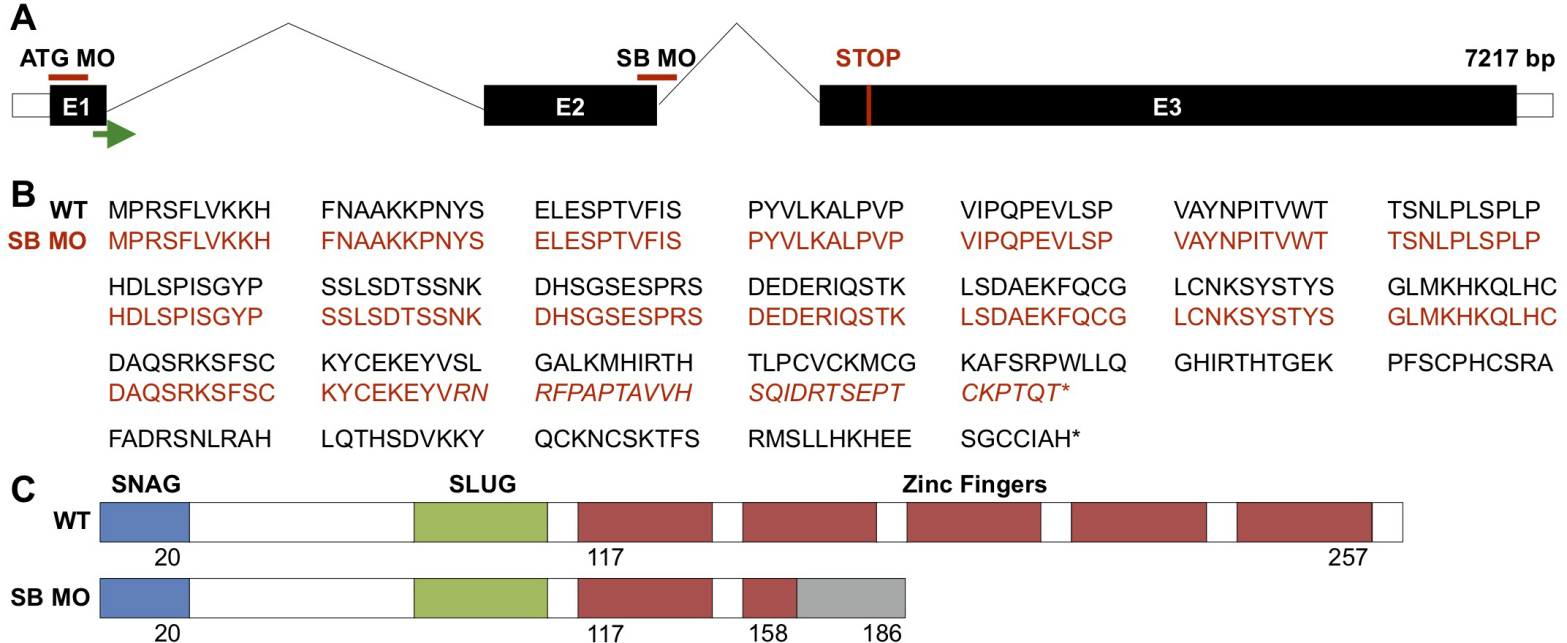
Predicted genomic and protein effects of *snai2* morpholinos. Location of the splice-blocking and translation-blocking morpholinos are indicated on a schematic of the *snai2* gene (A). The green arrow indicates the transcription start site, and the red line the endogenous stop codon. Predicted effects on the amino acid sequence (B) and protein structure (C) are shown for wild-type and SB morphant fish. 121 bp of exon 2 are lost due to the aberrant splicing event, leading to a truncation of the protein within the zinc-finger domain. Italicism of the amino acid sequence and the grey region of the structure indicate a region of missense amino acids prior to the early stop codon. E: exon; SB: splice-block; MO: morpholino.

**Fig 3:**
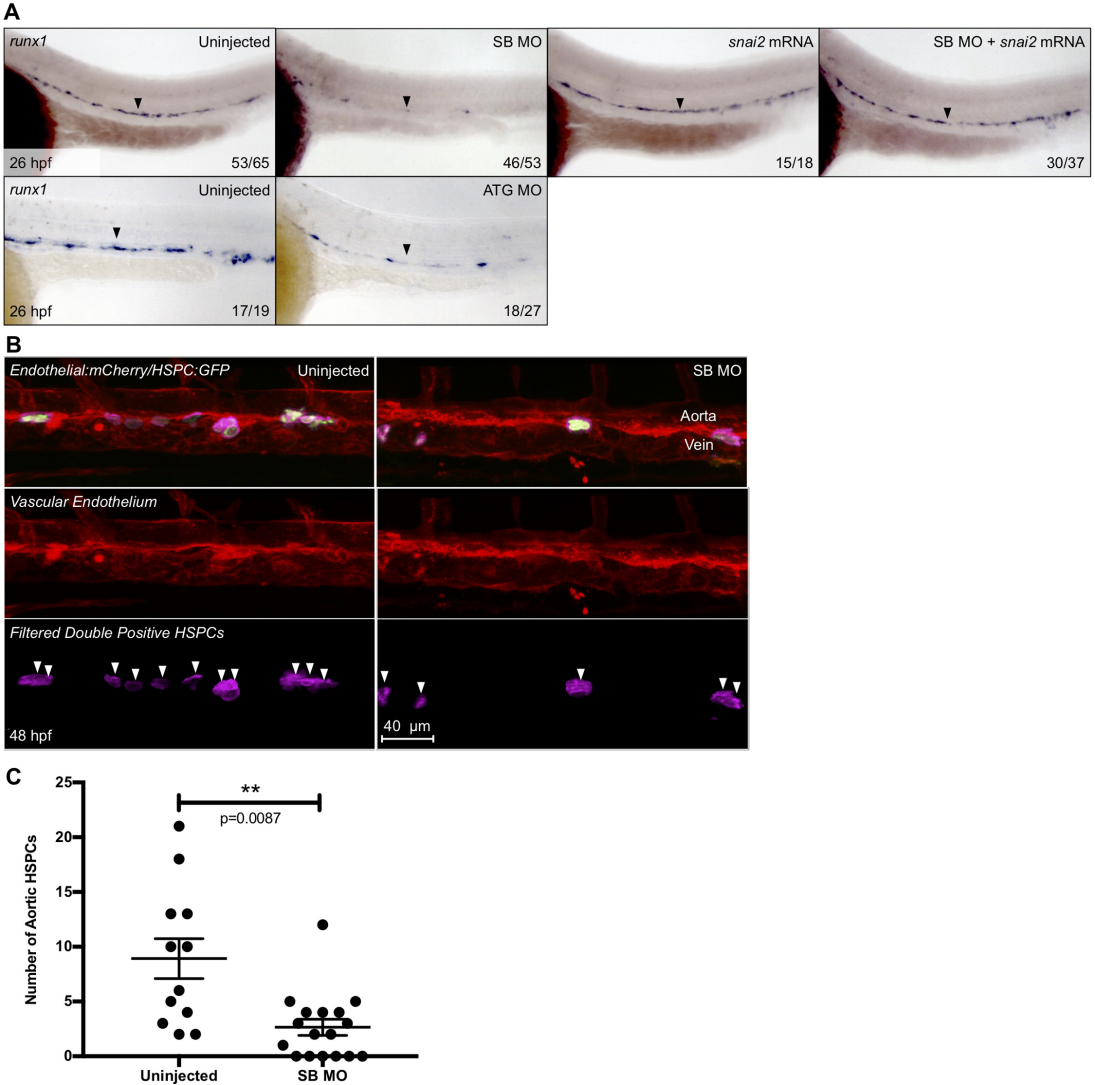
*Snai2* morphants display a strong defect in HSC specification. Expression of the HSC specification marker, *runx1*, was analyzed by *in situ* hybridization at ~26 hpf in embryos injected with SB MO, ATG MO, and their siblings (A). The effect of *snai2* mRNA injection was also analyzed both alone and when coinjected with SB MO. Black arrowheads point to the middle of the aortic *runx1* expression. Numbers in the lower right hand corner of each image depict the number of embryos with the phenotype pictured out of the total number of embryos assayed in each condition. *Tg(gata2b*:*Gal4/UAS*:*LA-GFP/kdrl*:*mCherry)* morphants and their siblings were imaged by confocal microscopy at 48 hpf, and Imaris imaging software was used to remove GFP signal outside of the vasculature (B). Pink coloration is indicative of double positive cells as filtered by the surfaces feature of Imaris. White arrowheads indicate separate putative HSPCs. Quantification for each fish was graphed and statistically analyzed by non-parametric *t*-test on Prism (C). Error bars are SEM.

We confirmed splicing defects using a previously reported splice blocking (SB) morpholino targeting the exon2-intron2 junction (Figs 2A and S1). The resulting misspliced gene product was sequenced to predict the amino acid sequence and protein structure, which indicated that the SB transcript should produce a protein truncated just beyond the first zinc finger (Fig 2B and 2C)[15,44]. The loss of the majority of the zinc-finger domains is expected to prevent DNA binding, and protein function. WISH for *runx1*, a marker of HSC commitment, indicated a decrease of HSCs in the dorsal aorta at 26 hpf, which could be rescued using an exogenous, SB-resistant *snai2* mRNA (Fig 3A). *Snai2* mRNA injected alone was not seen to affect expression of *runx1*. Furthermore, by 48 hpf, there was also a significant decrease of HSCs in the SB morphants, as assessed by confocal imaging of *gata2b* reporter embryos (Fig 3B and 3C).

The requirement for Snai2 in the derivation of endothelial cells was assessed using WISH for markers of the PLM (*fli1a*; S2 Fig) and the trunk vasculature (*fli1a*, *kdrl*; S2 Fig), which were normal. Primitive hematopoiesis appeared normal (*gata1*; S3 Fig). Additionally, the formation of the pronephros, which will develop into the adult hematopoietic niche, appeared normal (*cdh17*; S3 Fig).

These results were recapitulated using a translation blocking (ATG) morpholino (Fig 2A), which also resulted in a decrease in HSC specification at 26 hpf (Fig 3A). ATG morphants had a similar decrease in emerging HSPCs in Tg-HSC animals (S4 Fig). Levels of the hematopoietic marker *cmyb* in the caudal hematopoietic tissue at 48 hpf were also reduced, as previously reported in the SB morphants (S4 Fig). Taken together with the mRNA rescue and SB morpholino data, these results suggest that both morpholinos specifically target *snai2*. We completed our further studies using the SB morpholino alone.

### *Snai2* Morphants display sclerotome defects

**Fig 4:**
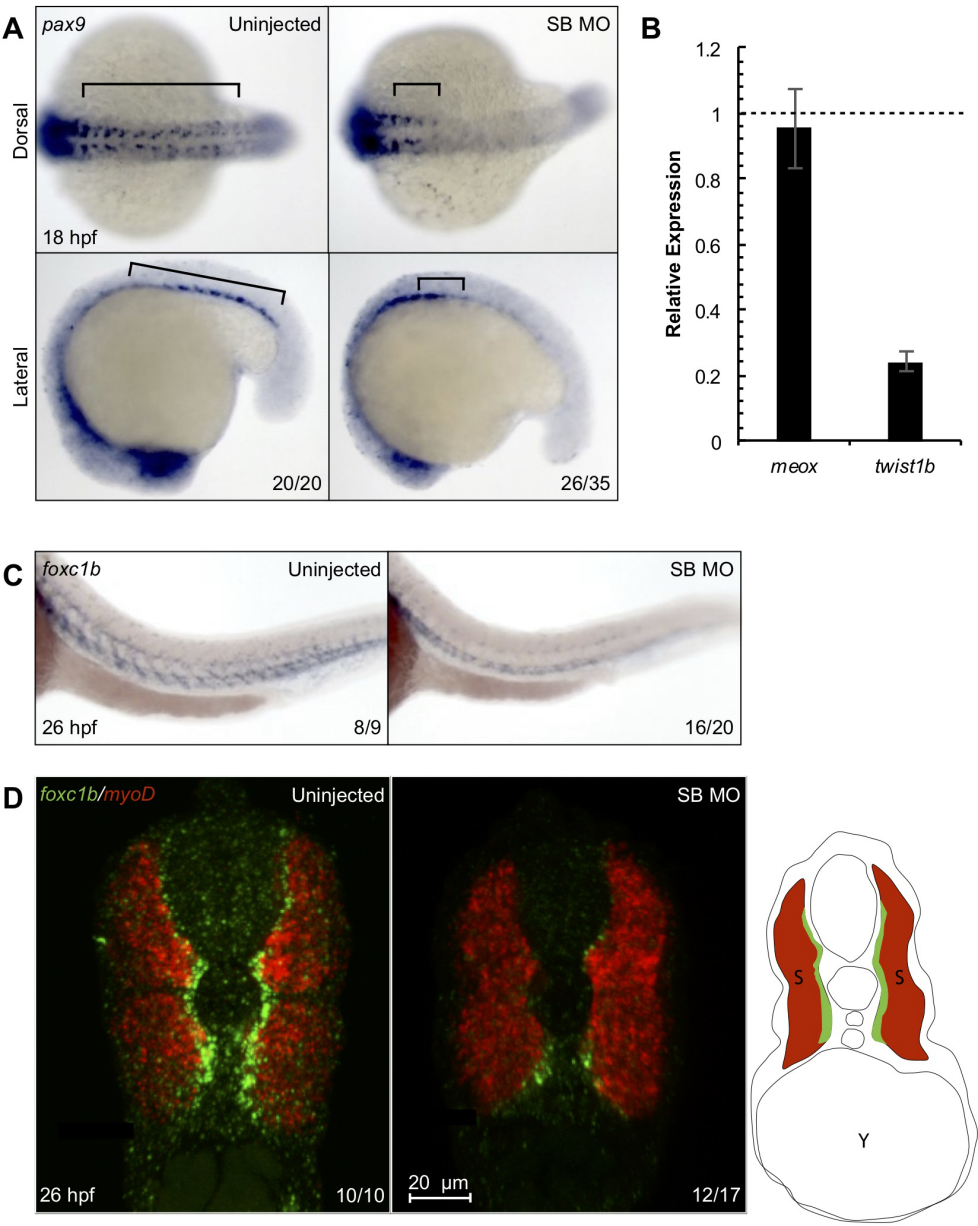
*Snai2* SB morphants display depletion of sclerotome markers. *pax9*, a marker of the sclerotome, was analyzed by WISH at 18 hpf (A). Black brackets highlight the difference in staining between morphants and siblings. qPCR on somitic, GFP^+^ cells sorted from morphant and control *Tg(actc1b*:*GFP)* embryos at ~17 hpf showed the sclerotome marker *twist1b* was decreased, while the pan-somitic marker remained normal in morphants (B). Wish for *foxc1b* showed clear diagonal expression in the anterior portion of the somites of uninjected embryos, while morphants lacked this distinctive stripe pattern (C). Error bars are calculated from technical replicates. Double fluorescent *in situ* hybridization for *foxc1b and myoD* (myogenic marker) followed by razor cutting for confocal analysis showed that while the muscle marker is consistent in both morphants and uninjected embryos, there is a notable decrease of positive staining for *foxc1b*, especially within the dorsal portion of the somites. A small schematic is provided to show greater detail of how embryos are oriented in Fig D. Numbers in the lower right-hand corner of each image depict the number of embryos with the phenotype pictured out of the total number of embryos assayed in each condition.

The combined observations of loss of early HSC specification in *snai2* morphants and high *snai2* expression in the ventral somites suggested that Snai2 may function in the sclerotome, which is known to be involved in HSC specification[10,11]. Indeed, *snai2* morphants lacked *pax9* (sclerotome marker) staining in their posterior somites, though *pax9* expression was unaffected in the most anterior somites (Fig 4A). Morphants also displayed a decrease in *twist1b* (sclerotome marker) transcripts by qPCR in sorted somitic cells, whereas levels of *meox* (pan-somitic marker) were unaffected (Fig 4B). This suggested that the sclerotome compartment of posterior somites is specifically affected by loss of *snai2*. Supporting this, the structure of the sclerotome was disrupted in SB MO embryos, as assessed by *foxc1b* (Fig 4C and 4D), while *myoD* (myogenic marker) expression was induced normally (Fig 4D), further supporting the specificity of Snai2 function. Altogether, these analyses indicate that *snai2* morphants have a defect in sclerotome formation that could directly affect the downstream specification and maintenance of HSCs.

### *Snai2* morphants display defective Notch signaling

**Fig 5:**
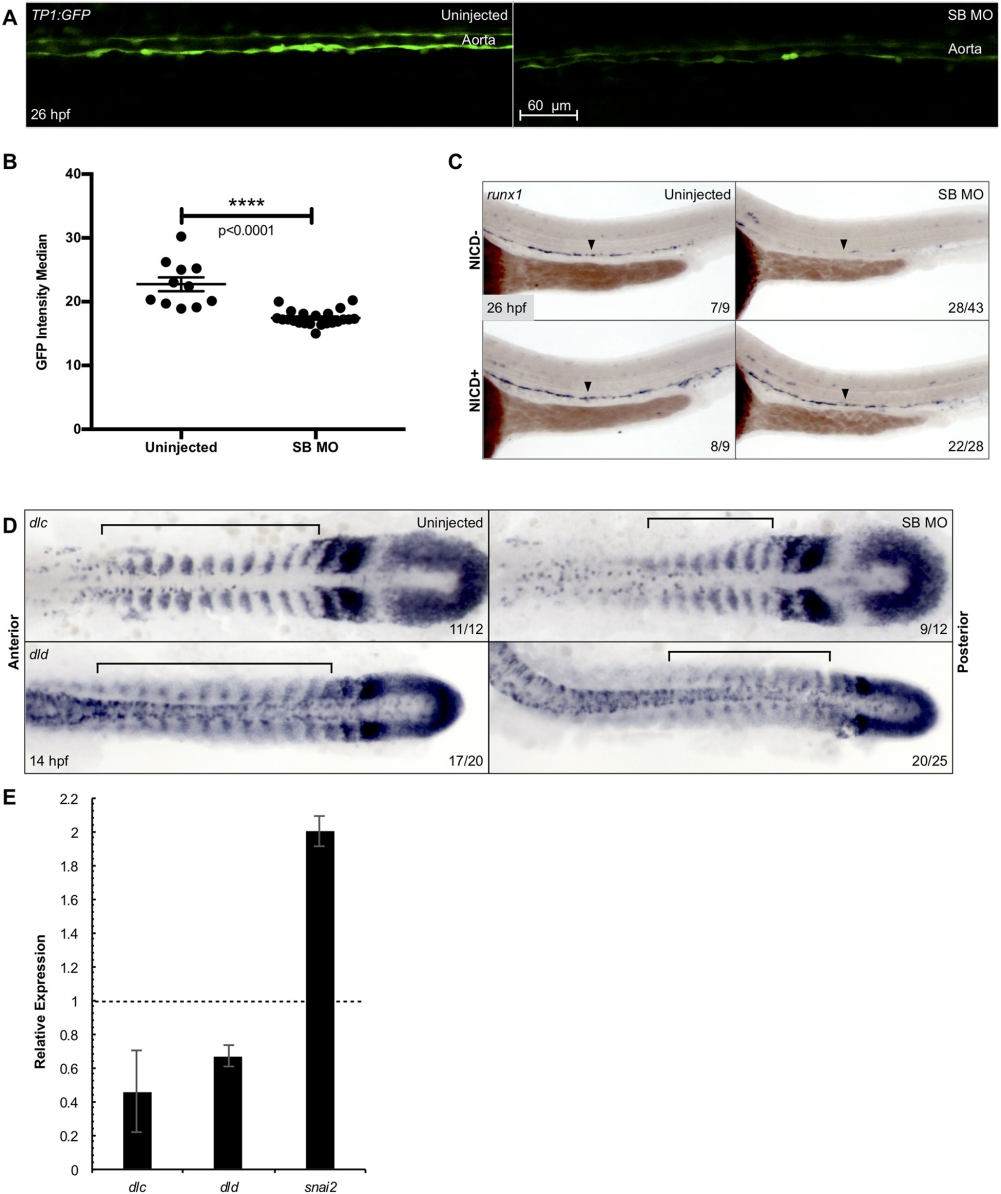
*Snai2* SB morphants have defective Notch signaling. Aortic Notch activity was assessed by confocal microscopy of the Notch reporter *Tg(TP1*:*GFP)* (A). Median fluorescence intensity was calculated by the surfaces feature of Imaris and was graphed and statistically analyzed by a non-parametric *t*-test on Prism (B). Error bars are SEM. Using a combination of *Tg(kdrl*:*miniGal4)* and *Tg(UAS*:*NICD-myc)*, we saw that ectopically activating Notch signaling within the endothelium was sufficient to rescue expression of the HSC marker *runx1* in morphant embryos (C). Black arrowheads point to the middle of the aortic *runx1* expression. Analysis by WISH displays that expression of the Notch ligands *dlc* and *dld* is decreased in morphants, especially within the more anterior somites (D). Black brackets are provided to highlight the differences in staining. This decrease was further confirmed by qPCR in somitic, GFP^+^ cells sorted from morphant *Tg(actc1b*:*GFP)* embryos as compared to uninjected siblings (E). *Snai2* relative expression is included for comparison, since the misspliced transcript is consistently elevated in SB MO injected embryos. Error bars are calculated from technical replicates. Numbers in the lower right-hand corner of each image depict the number of embryos with the phenotype pictured out of the total number of embryos assayed in each condition.

Notch signaling plays multiple essential roles in the specification of the HSC[50], and the ventral somites have been shown to be one source of this activity[7], which ultimately culminates in aortic Notch signaling during HSC emergence. Utilizing a transgenic Notch-activity reporter we determined that 26 hpf morphants lacked aortic Notch activity (Fig 5A and 5B). However, aortic identity was partially spared, as morphants had normal levels of aortic *dll4* and *dlc* (S5 Fig). This suggests that loss of *snai2* leads to loss of aortic Notch activity and HSCs. Indeed, endothelial-specific expression of the Notch1 intracellular domain was able to rescue loss of HSCs in *snai2* morphants (Figs 5C and S5), suggesting that Notch signaling lies downstream of Snai2 function.

The data above suggested that *snai2* deficient somites may be defective in Notch ligand production. In this regard, morphants were deficient for *dlc* and *dld* in the anterior somites (Fig 5D). These results were confirmed by qPCR on somitic cells (Fig 5E) and double fluorescent *in situ* for *dld* and *myoD* (S5 Fig). The SB morphants also have normal expression of the adherence protein *jam2a*, indicating that although Notch ligands are not presented properly from the somites, this tissue is still capable of closely interacting with the migrating PLM[7] (S5 Fig). Altogether, these data suggest that somitic *snai2* is required for *dlc* and *dld* expression, which is necessary for downstream Notch activity in HSC precursors.

### Generation of a Snail2 mutant zebrafish

**Fig 6:**
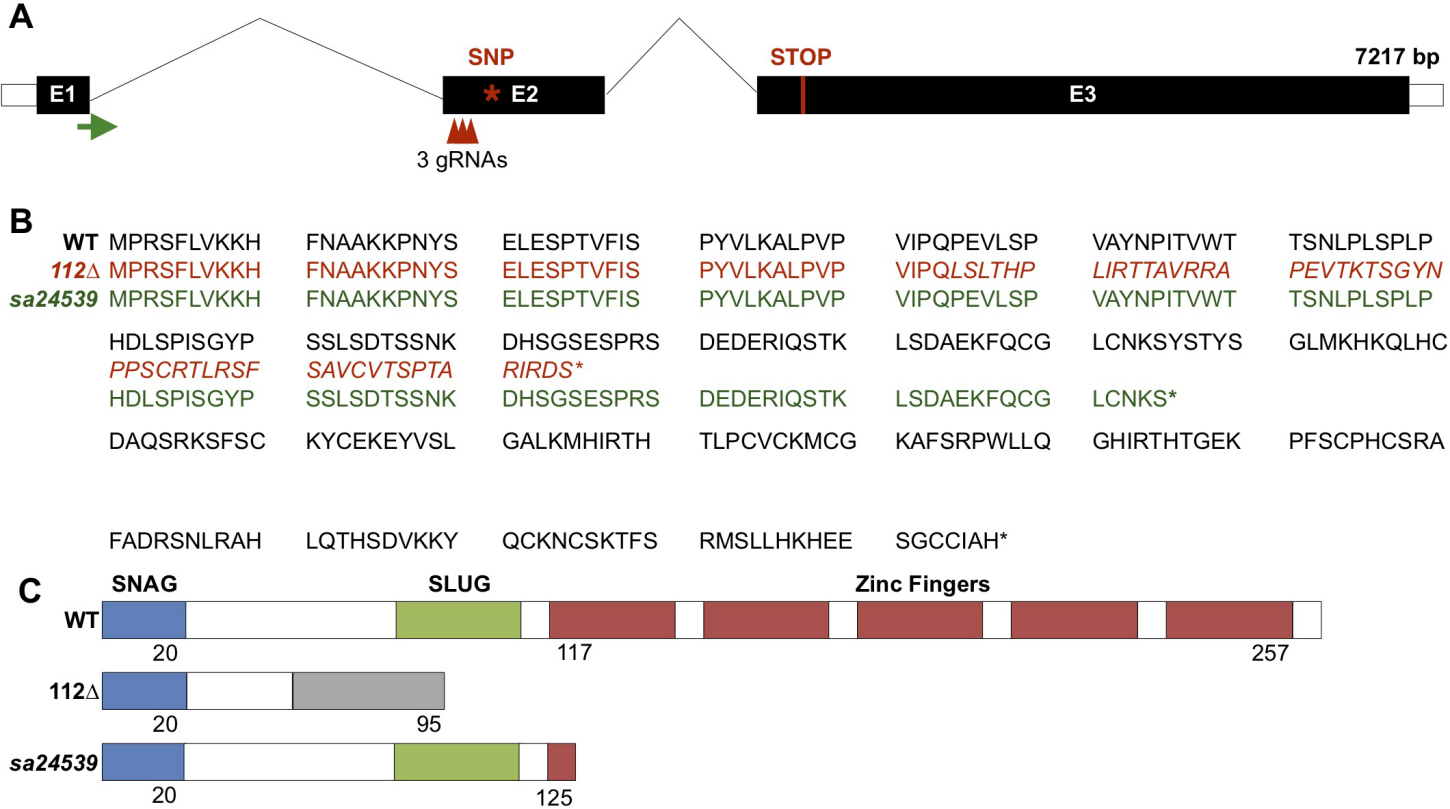
Predicted genomic and protein effects of the mutant *snai2* alleles. A schematic of the *snai2* gene displays where the three guide RNAs (gRNAs) were designed for CRISPR/Cas9 directed mutagenesis (red arrowheads) as well as the single nucleotide polymorphism (SNP) location in the mutagenesis derived *snai2*^*sa24539*^ allele (red *) (A). The endogenous stop codon is indicated by the red line in exon 3. Predicted effects on the amino acid sequence (B) and protein structure (C) of wild-type, *snai2*^*112*Δ^, and *snai2*^*sa24539*^ are depicted. For the *snai2*^*112*Δ^ allele, a 112 bp deletion within the beginning of exon 2 leads to a truncation prior to the zinc-finger domain. Italicism of the amino acid sequence and the grey region of the structure indicate a region of missense amino acids prior to the early stop codon. The *snai2*^*sa24539*^ allele SNP is a stop codon.

To test the effect of total Snail2 loss of function, we generated a mutant using CRISPR/Cas9 to induce a mutation in the second exon of the gene (Fig 6A). This mutation was analyzed by sequencing the resulting transcript. The zebrafish line possessed a 112 base pair deletion in the coding region, which is predicted to cause a frameshift, early stop codon, and truncated protein (Fig 6B). This deletion is easily detectable by PCR (S6 Fig). The truncated protein is predicted to lack function due to the loss of the SLUG recruitment domain and all DNA binding motifs (Fig 6C). We also obtained an ENU-derived mutant predicted to cause a truncated protein lacking most of the zinc finger domains (Fig 6A–6C).

### *Snai2*^*112Δ/112Δ*^ and *snai2* ^*sa24539/ sa24539*^ do not display defects in HSC specification or sclerotome formation

**Fig 7:**
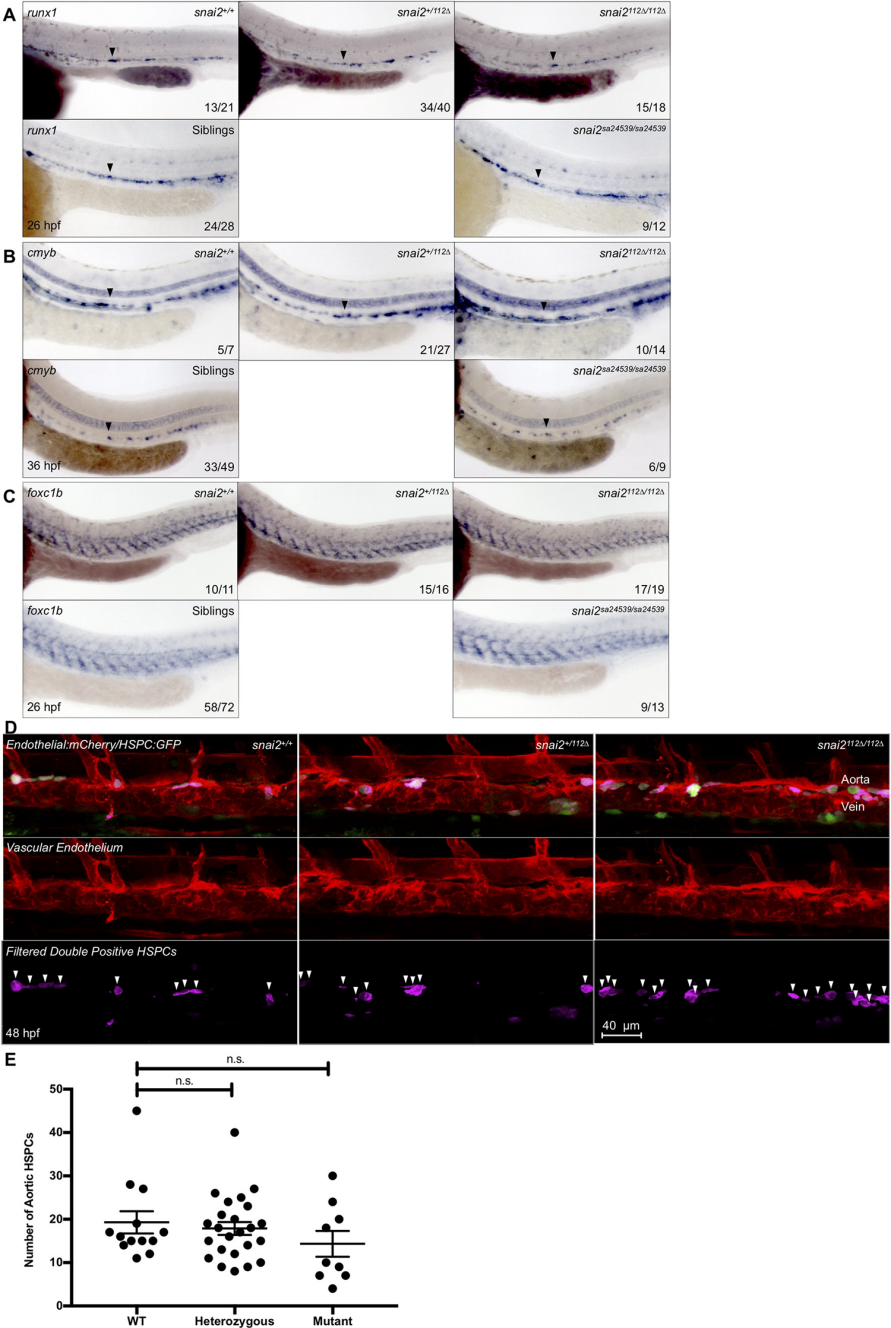
*Snai2*^*112Δ*^ and *snai2*^*sa24539*^ mutants have no embryonic defects in HSC or sclerotome formation. WISH analysis was performed on embryos derived from heterozygote in-crosses including probing for the hematopoietic markers *runx1* (A) and *cmyb* (B) and the sclerotome marker *foxc1b* (C). Wild-type, heterozygotes, and mutants are all included for the *Snai2*^*112Δ*^ allele since genotyping without sequencing was possible. For the *snai2*^*sa24539*^ allele, genotyping by PCR was sufficient to determine which embryos lacked the wild-type SNP, but not to distinguish wild-type from heterozygote. Thus, images are included for mutants versus “siblings”. For all markers, no obvious defect is detected. Black arrowheads point to the middle of the aortic *runx1* or *cmyb* expression. Numbers in the lower right-hand corner of each image depict the number of embryos with the phenotype pictured out of the total number of embryos assayed in each condition. HSC specification was also analyzed in *Tg(CD41*:*GFP/kdrl*:*mCherry)* fish on the *snai2*^*112Δ*^ background by confocal microscopy at 48 hpf, and Imaris imaging software was used to remove GFP signal outside of the vasculature (D). Pink coloration is indicative of double positive cells as filtered by the surfaces feature of Imaris. White arrowheads indicate separate putative HSPCs, and quantification for each fish was graphed and statistically analyzed by a non-parametric *t*-test on Prism (E). Error bars are SEM.

In contrast to morphants, there was no difference in the early specification marker *runx1* or the later hematopoietic marker *cmyb* in either *snai2*^*112Δ/112Δ*^ or *snai2* ^*sa24539/ sa24539*^ embryos (Fig 7A and 7B). The mutants also showed normal levels of the T-cell marker *rag1* (S6 Fig), suggesting normal embryonic HSPC differentiation capacity. Additionally, sclerotome formation was normal in both mutant lines, as assayed by expression of *foxc1b* (Fig 7C). Finally, there was no significant difference in emerging HSCs in Tg-HSC animals harboring the *snai2*^*112Δ/112Δ*^ mutation (Fig 7D and 7E). Altogether, these results indicated that embryonic hematopoiesis was normal in both *snai2* mutants.

### *Snai2*^*112Δ*^ mutants survive to adulthood with healthy whole kidney marrow

**Fig 8:**
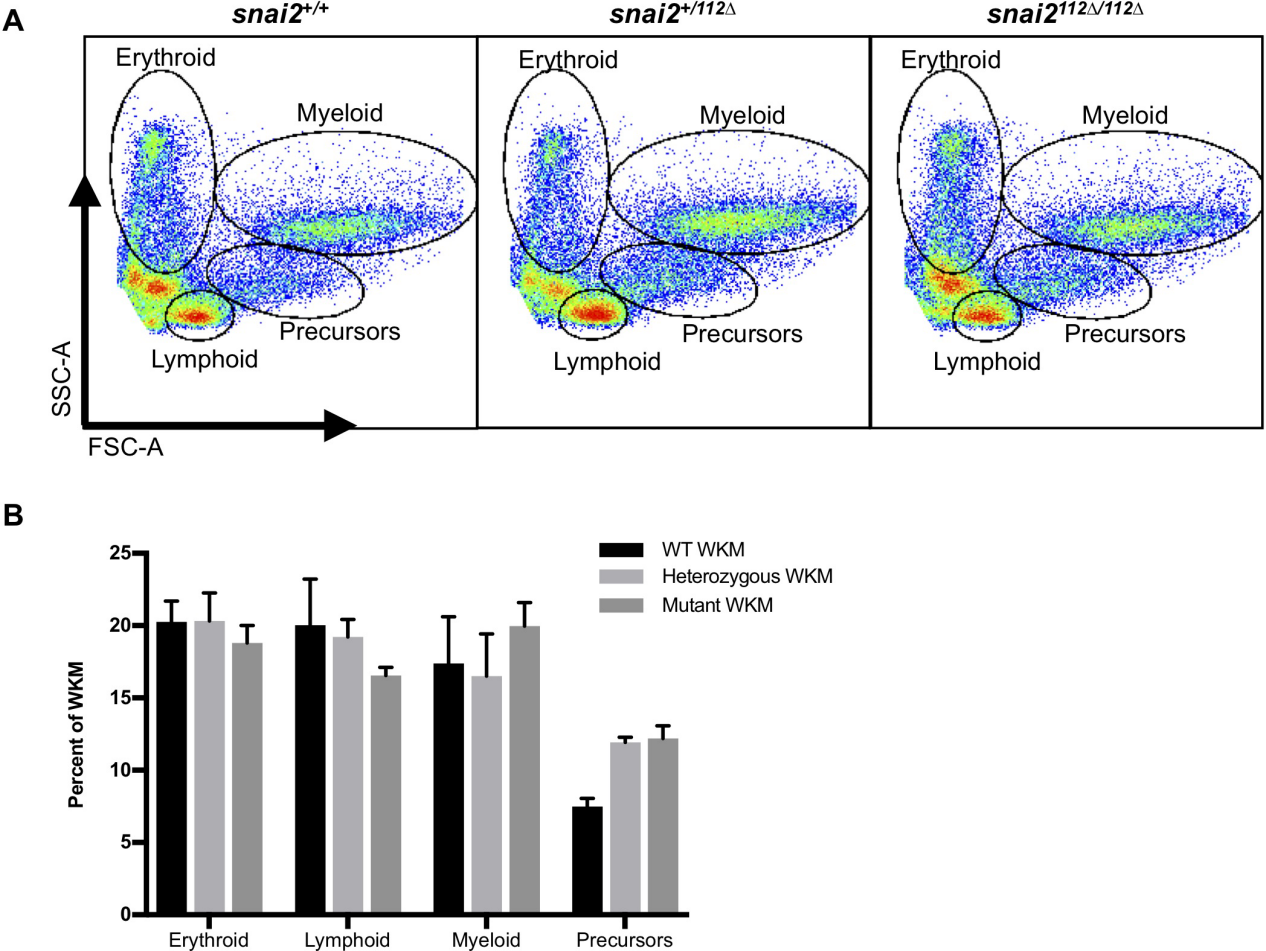
*Snai2*^*112Δ*^ mutant adult hematopoiesis appears normal. We analyzed the adult whole-kidney marrow (WKM) of 6-month-old *Snai2*^*112Δ*^ mutants and their siblings by flow cytometry. Analyzing the cells by forward and side-scatter (A) displayed no significant difference between the various hematopoietic populations via two-way ANOVA (B). Error bars are SEM.

The zebrafish kidney is the site of adult hematopoiesis, akin to mammalian bone marrow. Different cellular fractions were assayed by flow cytometry for size and granularity, where we found that wildtype, heterozygotes, and morphants had statistically similar cellular distributions, consistent with a lack of defects in embryonic hematopoiesis (Fig 8A and 8B). However, there is a slight trend towards less erythroid and lymphoid cells within the mutants, similar to the defects found in adult *snai2* mutant mice, which may suggest that this function is conserved. These mutant adults are also capable of breeding and producing healthy, viable progeny who grow to adulthood without incident.

### Probing morphant vs. mutant phenotype validity

**Fig 9:**
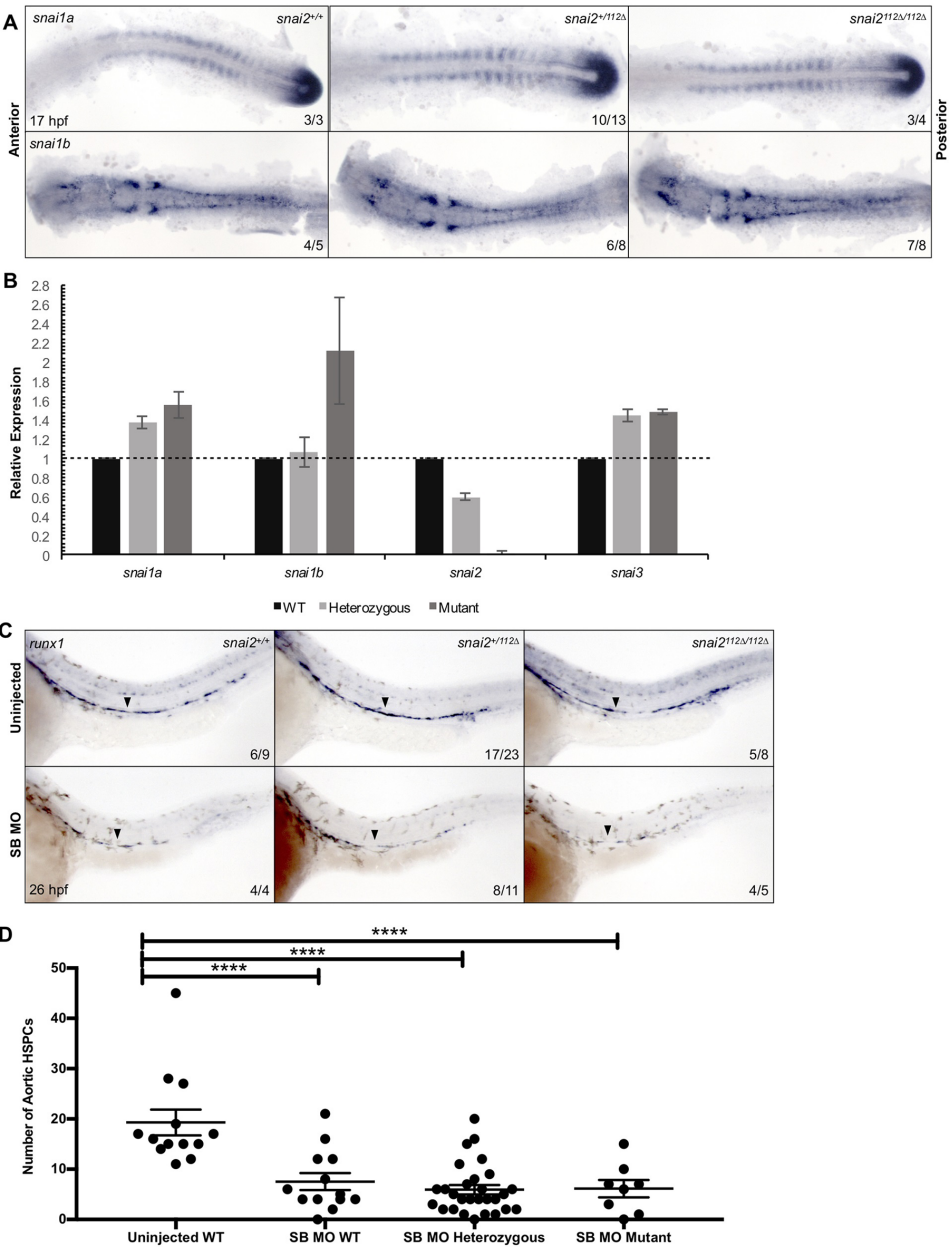
Preliminary investigation of morphant vs. mutant phenotype. WISH within 17 hpf embryos from an in-cross of *snai2*^*+/112Δ*^ displayed that the Snail family members *snai1a* and *snai1b* are not differentially expressed in mutant embryos as compared to heterozygote and wild-type siblings (A). However, qPCR in pooled embryonic trunks at 26 hpf showed a different trend: all 3 additional members of the Snail family show increased expression (B). This graph presents the average of two independent experiments in which embryonic heads were removed and genotyped, followed by pooling of trunks of the same genotype. Error bars are SD. *Snai2* reverse primer is designed within the mutant deletion, so transcript decrease reflects present of mutant transcript. We also observed the effect on emerging HSCs when the SB MO was injected into mutant embryos and their siblings by observing both WISH for *runx1* at 26 hpf (C), and the double positive population in *Tg(CD41*:*GFP/kdrl*:*mCherry)* embryos at 48 hpf. Double positive cells were filtered by the surfaces function on Imaris, quantified, and submitted to statistical testing by a non-parametric *t*-test on Prism (D). Error bars are SEM. By both analyses, HSC specification was affected by SB MO in all genetic backgrounds. Black arrowheads point to the middle of the aortic *runx1* expression. **** represents p<0.0001. WT: Wild-type.

Since recent studies have shown that permanent genomic disruption of a gene can lead to enhanced expression of similar genes[51], we examined the expression of all members of the Snail transcription factor family. WISH for both zebrafish orthologues of *snai1* at the time of PLM migration onset showed similar levels of expression in wildtype, heterozygous, and mutant embryos (Fig 9A). However, at 26 hpf, qPCR displayed a 1.5- to 2-fold increase of *snai1a*, *snai1b*, and *snai3* in the mutants (Fig 9B), suggesting some functional compensation may occur.

On the other hand, it has also been shown that some morpholinos have either off target or toxic effects on embryonic development that can masquerade as specific phenotypes. Therefore, we assayed whether injection of the SB MO was capable of causing a reduction in HSC specification in an embryo with only mutant *snai2*. Indeed, decreases in both *runx1* by WISH (Fig 9C) and in HSC emergence in a transgenic reporter line (Figs 9D and S6) were shown across all genotypes. *Snai2* ATG MO also decreased *runx1* expression across all genotypes (S6 Fig). Some morpholinos are known to cause phenotypes due to an increase in *p53* transcript[52]; however, qPCR on 26 hpf embryos showed only slight elevation, and *p53* mutation did not affect the number of HSPCs in SB morphants (S7 Fig).

## Discussion

In this study, we more closely analyzed the hematopoietic phenotype previously identified in *snai2* morphant zebrafish embryos and characterized two mutant zebrafish *snai2* alleles. Previously published data from *snai2* morphants had suggested that Snail2 plays a role in the etiology of HSCs, and our data from two independent morpholinos supported this. Additionally, we were able to rescue this HSC specification defect with *snai2* mRNA, indicating that the MO effect is specific. *Snai2* transcripts were highly expressed in the somites, a tissue known to be intimately involved in specification of the HSC. Furthermore, *snai2* morphants displayed defects in markers of the sclerotome and significant decreases of the somitic Notch ligands *dlc* and *dld*. Both of these defects are known to lead to problems with HSC formation, and in these morphants, *snai2* may play an upstream role in both sclerotome formation and Notch signaling. However, in two independent mutants of the *snai2* locus, we did not observe any significant defects in hematopoiesis. Indeed, *snai2*^*112Δ*^ mutants are capable of interbreeding and producing progeny that grow and thrive normally, indicating that maternal deposition of *snai2* transcript does not play a role in rescuing the mutant phenotype.

This discrepancy between morphant and mutant phenotype is a problem on the rise in zebrafish research. Multiple recent studies have described similar results focused on a variety of different genes ([25–29]), and there has been great debate on the topic of morpholino use. From one perspective, it seems that the potential off target or toxic effects of these oligonucleotides outweighs their utility as a tool for genetic knockdown; however, an in depth study has also shown that deleterious mutations can induce complex genetic compensation that can obscure the role of a factor in a given process[51]. In that case, a finely tuned knockdown of gene expression allows us to decipher a factor’s role, while a knockout may induce too much genetic compensation to deconvolute this. Both possibilities must be equally weighed, and great care must be taken when characterizing both new morpholinos and mutant alleles. New guidelines are now emerging to help deal with this growing issue[30]; however, the reality is that there are endless studies that now should be revisited to further asses genetic function.

The *snai2* morpholinos were capable of inducing a defect in HSC specification in the *snai2*^*112Δ*^ mutant, which strongly argues that the morpholino has an effect on either overall toxicity, or affects a target other than *snai2*, assuming the mutant does indeed cause loss of function. Additionally, while analysis of the *snai2* null mouse had shown a variety of effects on adult hematopoiesis, no defect in HSC specification or emergence has been identified. Finally, the increase of *p53* transcripts seen in the *snai2* SB morphants could indicate a problem with overall toxicity.

On the other hand, we present strong data that the morpholino knockdown of *snai2* is specific. The effect on HSC formation is specific: mRNA overexpression is capable of rescuing the *runx1* phenotype, and the second morpholino phenocopies the first. The overall development of morphant embryos is minimally affected, and structures including the aorta, cardinal vein, pronephros and most of the somite are unaffected. Finally, the Notch deficiency seen in the *snai2* morphants, is rescuable by overexpression of NICD in the vascular endothelium, again indicating a specific effect.

In further support of the morphant phenotype, the *snai2*^*112Δ*^ mutants show elevated *snai1a*, *snai1b*, and *snai3* transcripts, which could indicate some level of genetic compensation, and that one of the other factors could be taking on the role of *snai2* when absent. It is also formally possible that there are unknown factors compensating for this genetic loss of function. Further testing within the mutant would be essential to determine whether or not this is the case, including a much deeper probing of the mutant exome. It would also be interesting to create stable mutant forms of the other Snail family members, and then cross them appropriately to the Snail2 mutant fish. Previous studies have shown genetic redundancy by such an approach when a morpholino and mutant phenotype did not agree[53]. We performed a preliminary test of this redundancy by injecting *snai1a* morpholino into the *snai2*^*112Δ*^ mutants and their siblings; however, *runx1* expression was not strongly affected(S6 Fig). It may be the case that a second factor is still providing compensation, or that Snail1a in particular does not function redundantly with Snail2.

Another possibility is that these mutant alleles do not generate a true loss of function. The effects on protein translation can be predicted *in silico*; however, due to the absence of a zebrafish specific antibody to Snail2, it is impossible to know if these animals have generated a partially functional truncated protein, perhaps due to an alternative start site downstream of these mutations. One such potential start site would produce a truncated protein consisting of only zinc-finger domains. Moreover, if this protein was functional, the SB MO would be able to disrupt this transcript and protein, which could explain the HSC defect caused by the morpholino in the *snai2*^*112Δ*^ mutant embryos. However, studies have shown that *snai2* activity is largely dependent on the N-terminal SNAG and SLUG domains [54], so the truncated protein may not be sufficient to drive this function. Although protein analysis of Snail2 would be ideal to address these possibilities, testing of an available Snail2 antibody showed positive detection of murine, cardiac Snail2 was possible, but failed to detect zebrafish snail2 when overexpressed in HEK-293T cells (S8 Fig).

Finally, it is possible that the morpholino results in a dominant-negative protein which causes a negative effect on HSC specification. The misspliced transcript does not undergo nonsense mediated decay and accumulates, as shown by qPCR.

In summary, our results provide further evidence that understanding genetic function must be approached simultaneously with multiple approaches. The data presented here supports that the *snai2* morphant phenotype could be due to an off target or toxic event; however, this could also be due to reasons explained above, and further studies are necessary to deconvolute this problem.

## Supporting information

S1 Fig FACS and Morpholino controls. When double positive cells were sorted from *Tg(CD41*:*GFP/kdrl*:*mCherry)* embryos at 48 hpf, single positive mCherry only cells were also purified and qPCR performed with the same panel of genes: hematopoietic marker *cmyb*, endothelial marker *kdrl*, and *snai2* (A). As expected, *kdrl* was extremely elevated, while *cmyb* was decreased as compared to the rest of the embryo. *Snai2* is present, but extremely down regulated. Error bars are calculated from technical replicates. We confirmed efficacy of the SB MO by injecting into embryos and collecting a pool of embryos at 26 hpf. After RT-PCR was performed on a portion of the *snai2* transcript. 100% of the transcript appears to be the shortened length caused by the error in splicing (B).
(TIFF)

S2 Fig Migration of the PLM and vascular cord formation is normal in SB morphants. WISH was performed on embryos injected with SB MO and their uninjected siblings to investigate migration of the PLM and formation of the vascular cord. We analyzed *fli1a*, a gene actively expressed in the PLM as well as in the fully formed vasculature, as well as *kdrl*, a marker strong in the fully formed vasculature. At 14 and 18 hpf, *fli1a* staining showed normal formation of the PLM and timely migration to the midline (A). At 26 hpf, the vascular cord and caudal hematopoietic tissue appear largely normal by both *fli1a* and *kdrl* staining; however, the intersomitic vessels seem to have some trouble sprouting dorsally (B). Numbers in the lower right-hand corner of each image depict the number of embryos with the phenotype pictured out of the total number of embryos assayed in each condition.
(TIF)

S3 Fig Primitive hematopoiesis and pronephros formation are unaffected in SB morphants. In order to observe other tissues involved in embryonic hematopoiesis, we assayed primitive hematopoiesis by WISH for the early erythroid marker *gata1* (A). SB morphants appeared to have normal primitive hematopoiesis initiation. We also observed formation of the pronephros, which will develop to be the adult HSC niche, by WISH for *cdh17* (B). Pronephric formation appeared normal in SB morphants. Numbers in the lower right-hand corner of each image depict the number of embryos with the phenotype pictured out of the total number of embryos assayed in each condition.
(TIF)

S4 Fig Further analysis of ATG MO hematopoietic phenotype. ATG MO embryos were subjected to WISH for the hematopoietic marker *cmyb* at 48 hpf (A). The caudal hematopoietic tissue of morphant embryos showed a distinct reduction of *cmyb* staining as compared to their uninjected siblings. The morpholino was also injected into *Tg(CD41*:*GFP/kdrl*:*mCherry)* embryos and double positive fish were imaged via confocal microscopy at 48 hpf and Imaris imaging software was used to remove GFP signal outside of the vasculature (B). The surfaces feature of Imaris was utilized to quantify double positive cells (shown here in pink), and the resulting data was graphed and statistically analyzed by a non-parametric *t*-test on Prism (C). Error bars are SEM. There was a small, but significant decrease in the number of HSPCs in the ATG morphant fish. Numbers in the lower right-hand corner of each image depict the number of embryos with the phenotype pictured out of the total number of embryos assayed in each condition.
(TIFF)

S5 Fig Further notch and somitic morphant data. In order to show not all Notch ligand expression was affected in *snai2* SB morphants, we analyzed aortic expression of *dll4* and *dlc* by WISH at 26 hpf (A). SB morphants showed normal levels of both ligands supporting that the aorta is specified correctly. The presence of the Notch intracellular domain in *Tg(UAS*:*NICD-myc)* embryos can be assayed by immunohistochemistry for the myc tag, fused to the NICD. Representative images were taken of positive and negative staining present when the transgenic was crossed to the *Tg(kdrl*:*miniGal4)* (B). Staining is visible in the dorsal aorta and caudal vein, as well as quite strongly in the caudal hematopoietic tissue of Gal4^+^/NICD^+^ embryos. Double fluorescent *in situ* for *dld* and *myoD* was performed in SB morphants and their siblings at 14 hpf, and the results imaged by confocal microscopy (C). Representative images show that morphant embryos have decreased somitic *dld* staining, especially within the more anterior somites. *myoD* in the same somites was expressed normally. We also analyzed *jam2a* expression by WISH (D), since not only is this gene expressed within the somites, but it has been shown to be essential for notch signal transduction to the migrating PLM. SB morphants showed normal expression of *jam2a*. Numbers in the lower right-hand corner of each image depict the number of embryos with the phenotype pictured out of the total number of embryos assayed in each condition.
(TIF)

S6 Fig *Snai2*^*112Δ*^ further analysis. A representative gel image shows the different banding pattern observed when genotyping embryos from a *snai2*^*+/112Δ*^ in-cross (A). In order to assess later stages of embryonic hematopoiesis, we assessed expression of the T-cell marker, *rag1*, in 4 dpf embryos (B). Wild-types, heterozygotes, and mutants all showed normal *rag1* staining. When *snai2* mutants were analyzed on the *Tg(CD41*:*GFP/kdrl*:*mCherry)* background, we simultaneously injected a portion of the clutch analyzed with SB MO. These embryos were imaged via confocal microscopy and Imaris imaging software was used to remove GFP signal outside of the vasculature (C) alongside their uninjected siblings shown in Fig 7D. Quantification is shown in Fig 9D. Additionally, expression of the HSC specification marker, *runx1*, was analyzed by in situ hybridization at ~26 hpf in embryos injected with *snai2* ATG MO, *snai1a* morpholino (MO), and their siblings. Black arrowheads point to the middle of the aortic runx1 expression. Numbers in the lower right-hand corner of each image depict the number of embryos with the phenotype pictured out of the total number of embryos assayed in each condition.
(TIF)

S7 Fig SB MO causes an increase in *p53* transcript, but loss of *p53* does not rescue hematopoietic phenotype. The potential of toxicity caused by the SB MO was analyzed by qPCR for *p53* in morphant and uninjected pooled embryos at 26 hpf (A). Via this analysis, we saw that indeed *p53* transcript was increased as compared to uninjected siblings. We also show *snai2* transcript levels, as they are consistently increased in SB MO injected embryos. SB MO was then injected into embryos derived from a *p53*^*+/-*^ in-cross, and the embryos analyzed for the hematopoietic markers *rag1* and *cmyb*. The loss of *p53* did not appear to rescue the morphant phenotype of decreased levels of both genes. Black arrowheads indicate the general position of the thymus.
(TIF)

S8 Fig Western blot analysis shows available Snail2 antibody detects murine, but not zebrafish Snail2. Protein lysates from HEK-293T cells transfected with the zebrafish Snail proteins N-terminally tagged with green fluorescent protein (GFP) were analyzed via western blot for both GFP and Snail2. Mouse heart lysate was also run as a positive control for Snail2 presence. Protein size is indicated to the left of each blot. Lanes are labeled with the appropriate number representing the zebrafish Snail family member.
(TIFF)
