# Supplementary figures and images for: Zebrafish snai2 mutants fail to phenocopy morphant phenotypes
Source: PLoS One. 2018 Sep 12;13(9):e0202747. doi: 10.1371/journal.pone.0202747 (PMC6135377; doi:10.1371/journal.pone.0202747)

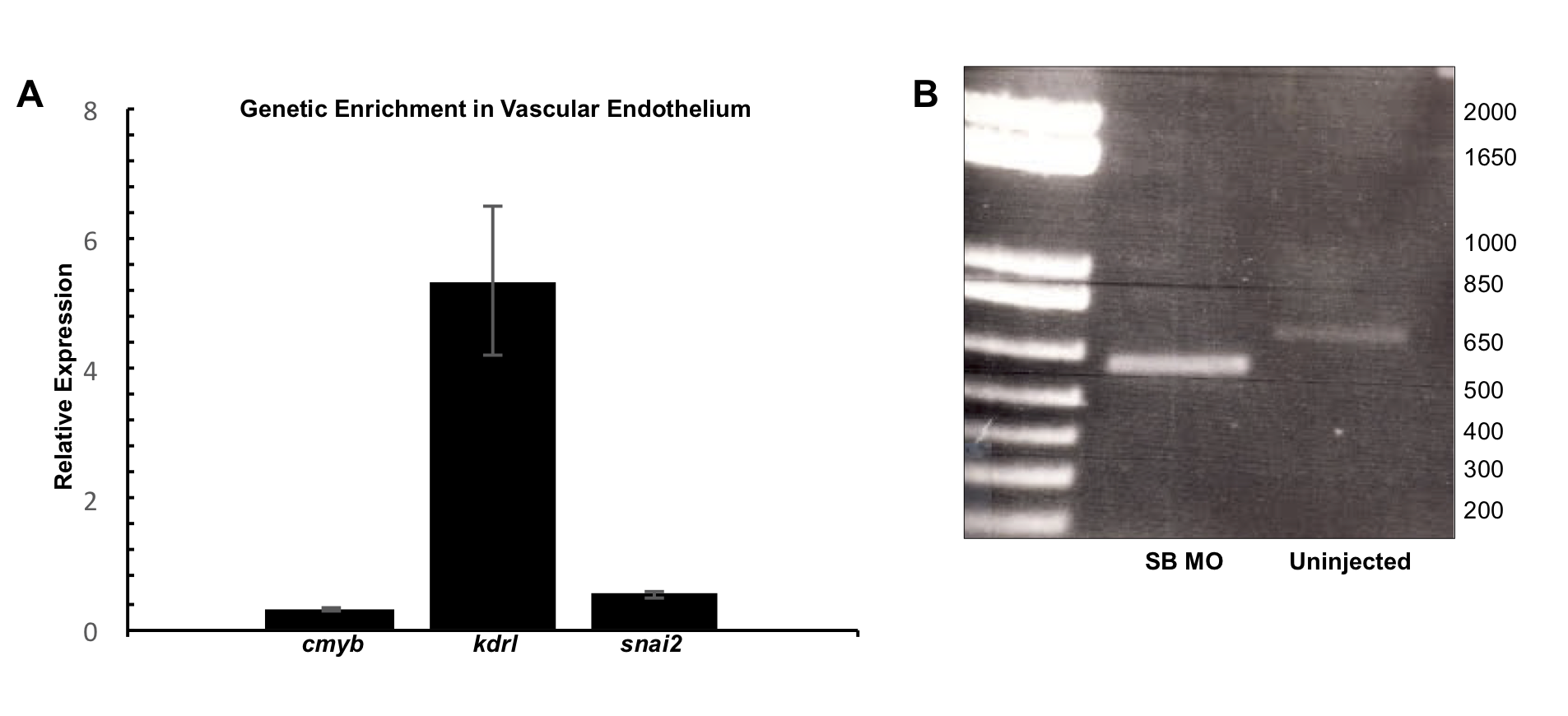

Supplement: S1 Fig — When double positive cells were sorted from Tg(CD41:GFP/kdrl:mCherry) embryos at 48 hpf, single positive mCherry only cells were also purified and qPCR performed with the same panel of genes: hematopoietic marker cmyb, endothelial marker kdrl, and snai2 (A). As expected, kdrl was extremely elevated, while cmyb was decreased as compared to the rest of the embryo. Snai2 is present, but extremely down regulated. Error bars are calculated from technical replicates. We confirmed efficacy of the SB MO by injecting into embryos and collecting a pool of embryos at 26 hpf. After RT-PCR was performed on a portion of the snai2 transcript. 100% of the transcript appears to be the shortened length caused by the error in splicing (B). (TIFF) [file pone.0202747.s001.tiff]

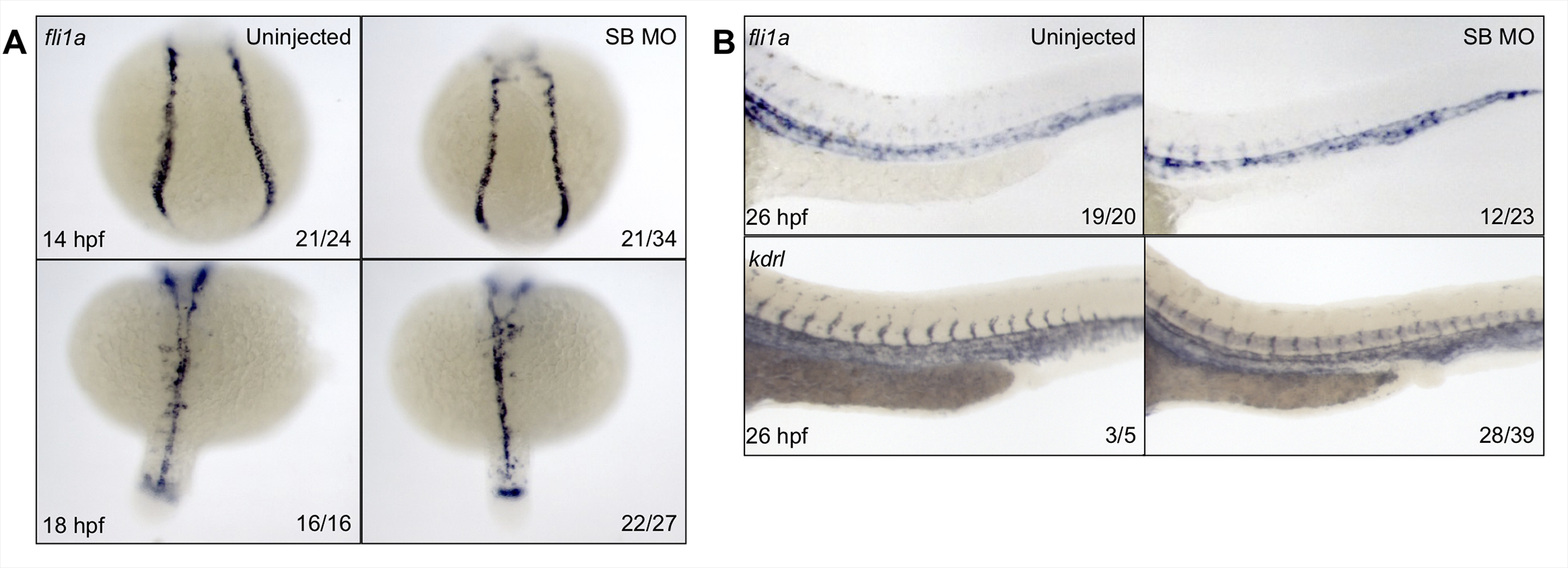

Supplement: S2 Fig — WISH was performed on embryos injected with SB MO and their uninjected siblings to investigate migration of the PLM and formation of the vascular cord. We analyzed fli1a, a gene actively expressed in the PLM as well as in the fully formed vasculature, as well as kdrl, a marker strong in the fully formed vasculature. At 14 and 18 hpf, fli1a staining showed normal formation of the PLM and timely migration to the midline (A). At 26 hpf, the vascular cord and caudal hematopoietic tissue appear largely normal by both fli1a and kdrl staining; however, the intersomitic vessels seem to have some trouble sprouting dorsally (B). Numbers in the lower right-hand corner of each image depict the number of embryos with the phenotype pictured out of the total number of embryos assayed in each condition. (TIF) [file pone.0202747.s002.tif]

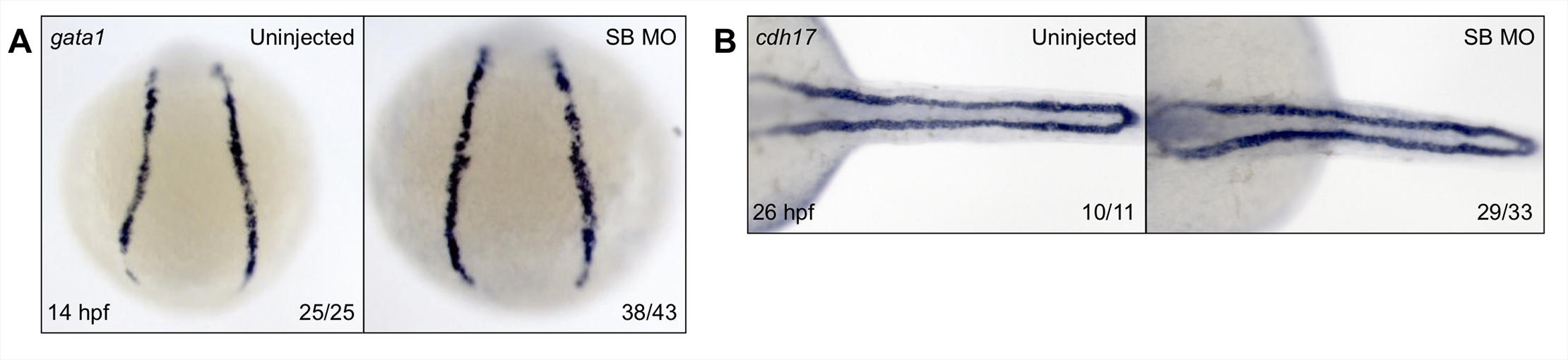

Supplement: S3 Fig — In order to observe other tissues involved in embryonic hematopoiesis, we assayed primitive hematopoiesis by WISH for the early erythroid marker gata1 (A). SB morphants appeared to have normal primitive hematopoiesis initiation. We also observed formation of the pronephros, which will develop to be the adult HSC niche, by WISH for cdh17 (B). Pronephric formation appeared normal in SB morphants. Numbers in the lower right-hand corner of each image depict the number of embryos with the phenotype pictured out of the total number of embryos assayed in each condition. (TIF) [file pone.0202747.s003.tif]

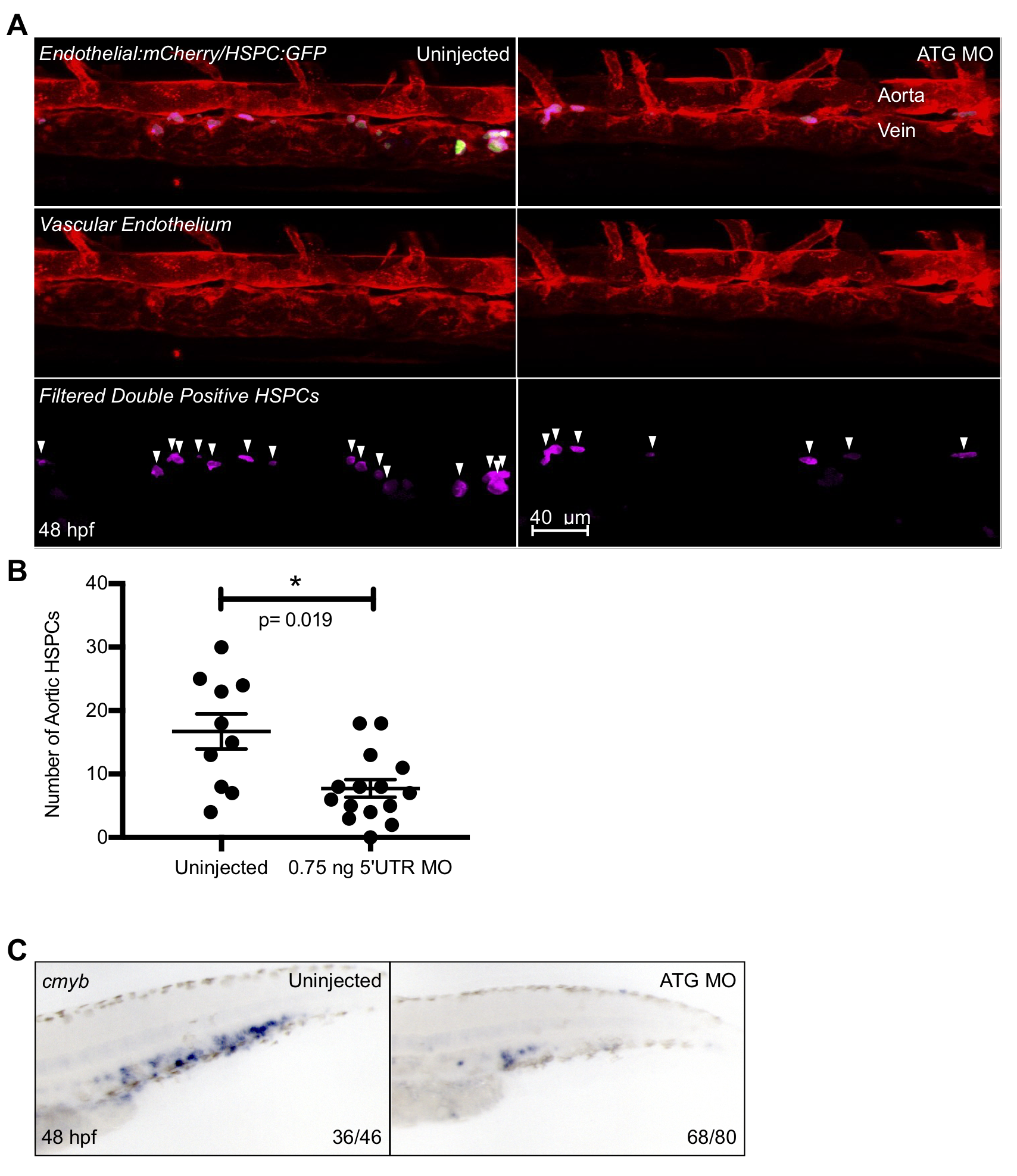

Supplement: S4 Fig — ATG MO embryos were subjected to WISH for the hematopoietic marker cmyb at 48 hpf (A). The caudal hematopoietic tissue of morphant embryos showed a distinct reduction of cmyb staining as compared to their uninjected siblings. The morpholino was also injected into Tg(CD41:GFP/kdrl:mCherry) embryos and double positive fish were imaged via confocal microscopy at 48 hpf and Imaris imaging software was used to remove GFP signal outside of the vasculature (B). The surfaces feature of Imaris was utilized to quantify double positive cells (shown here in pink), and the resulting data was graphed and statistically analyzed by a non-parametric t-test on Prism (C). Error bars are SEM. There was a small, but significant decrease in the number of HSPCs in the ATG morphant fish. Numbers in the lower right-hand corner of each image depict the number of embryos with the phenotype pictured out of the total number of embryos assayed in each condition. (TIFF) [file pone.0202747.s004.tiff]

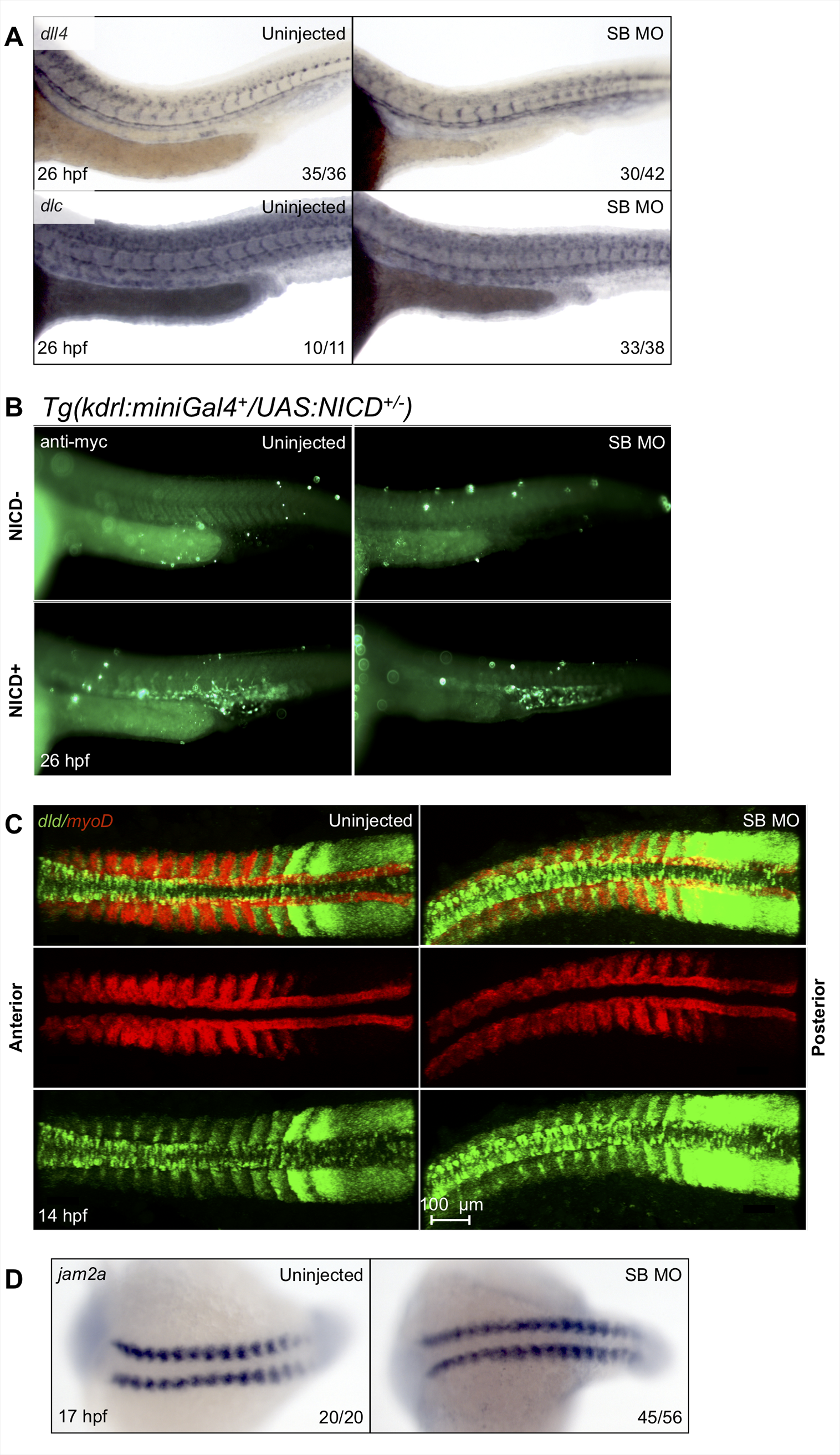

Supplement: S5 Fig — In order to show not all Notch ligand expression was affected in snai2 SB morphants, we analyzed aortic expression of dll4 and dlc by WISH at 26 hpf (A). SB morphants showed normal levels of both ligands supporting that the aorta is specified correctly. The presence of the Notch intracellular domain in Tg(UAS:NICD-myc) embryos can be assayed by immunohistochemistry for the myc tag, fused to the NICD. Representative images were taken of positive and negative staining present when the transgenic was crossed to the Tg(kdrl:miniGal4) (B). Staining is visible in the dorsal aorta and caudal vein, as well as quite strongly in the caudal hematopoietic tissue of Gal4+/NICD+ embryos. Double fluorescent in situ for dld and myoD was performed in SB morphants and their siblings at 14 hpf, and the results imaged by confocal microscopy (C). Representative images show that morphant embryos have decreased somitic dld staining, especially within the more anterior somites. myoD in the same somites was expressed normally. We also analyzed jam2a expression by WISH (D), since not only is this gene expressed within the somites, but it has been shown to be essential for notch signal transduction to the migrating PLM. SB morphants showed normal expression of jam2a. Numbers in the lower right-hand corner of each image depict the number of embryos with the phenotype pictured out of the total number of embryos assayed in each condition. (TIF) [file pone.0202747.s005.tif]

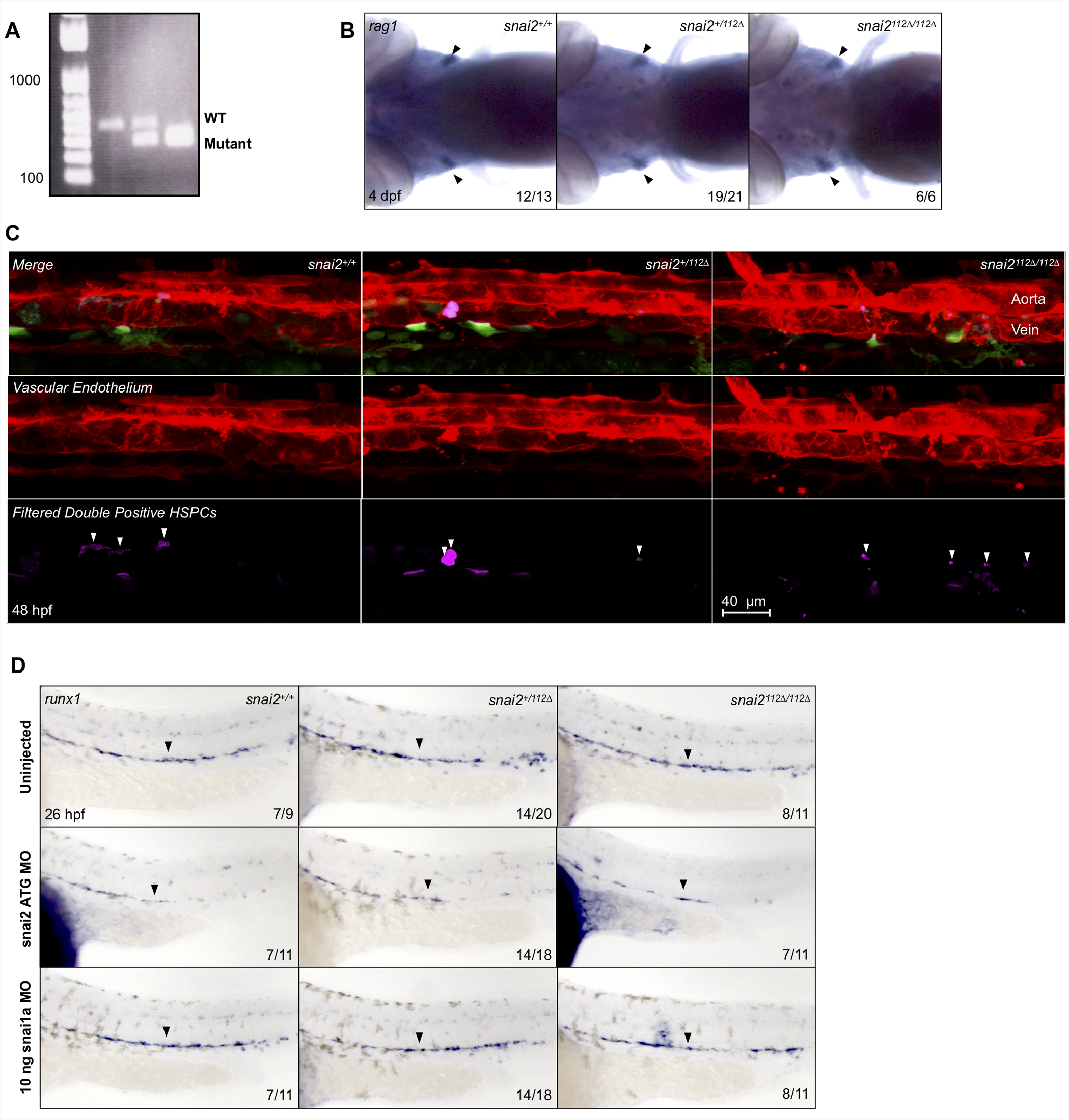

Supplement: S6 Fig — A representative gel image shows the different banding pattern observed when genotyping embryos from a snai2+/112Δ in-cross (A). In order to assess later stages of embryonic hematopoiesis, we assessed expression of the T-cell marker, rag1, in 4 dpf embryos (B). Wild-types, heterozygotes, and mutants all showed normal rag1 staining. When snai2 mutants were analyzed on the Tg(CD41:GFP/kdrl:mCherry) background, we simultaneously injected a portion of the clutch analyzed with SB MO. These embryos were imaged via confocal microscopy and Imaris imaging software was used to remove GFP signal outside of the vasculature (C) alongside their uninjected siblings shown in Fig 7D. Quantification is shown in Fig 9D. Additionally, expression of the HSC specification marker, runx1, was analyzed by in situ hybridization at ~26 hpf in embryos injected with snai2 ATG MO, snai1a morpholino (MO), and their siblings. Black arrowheads point to the middle of the aortic runx1 expression. Numbers in the lower right-hand corner of each image depict the number of embryos with the phenotype pictured out of the total number of embryos assayed in each condition. (TIF) [file pone.0202747.s006.tif]

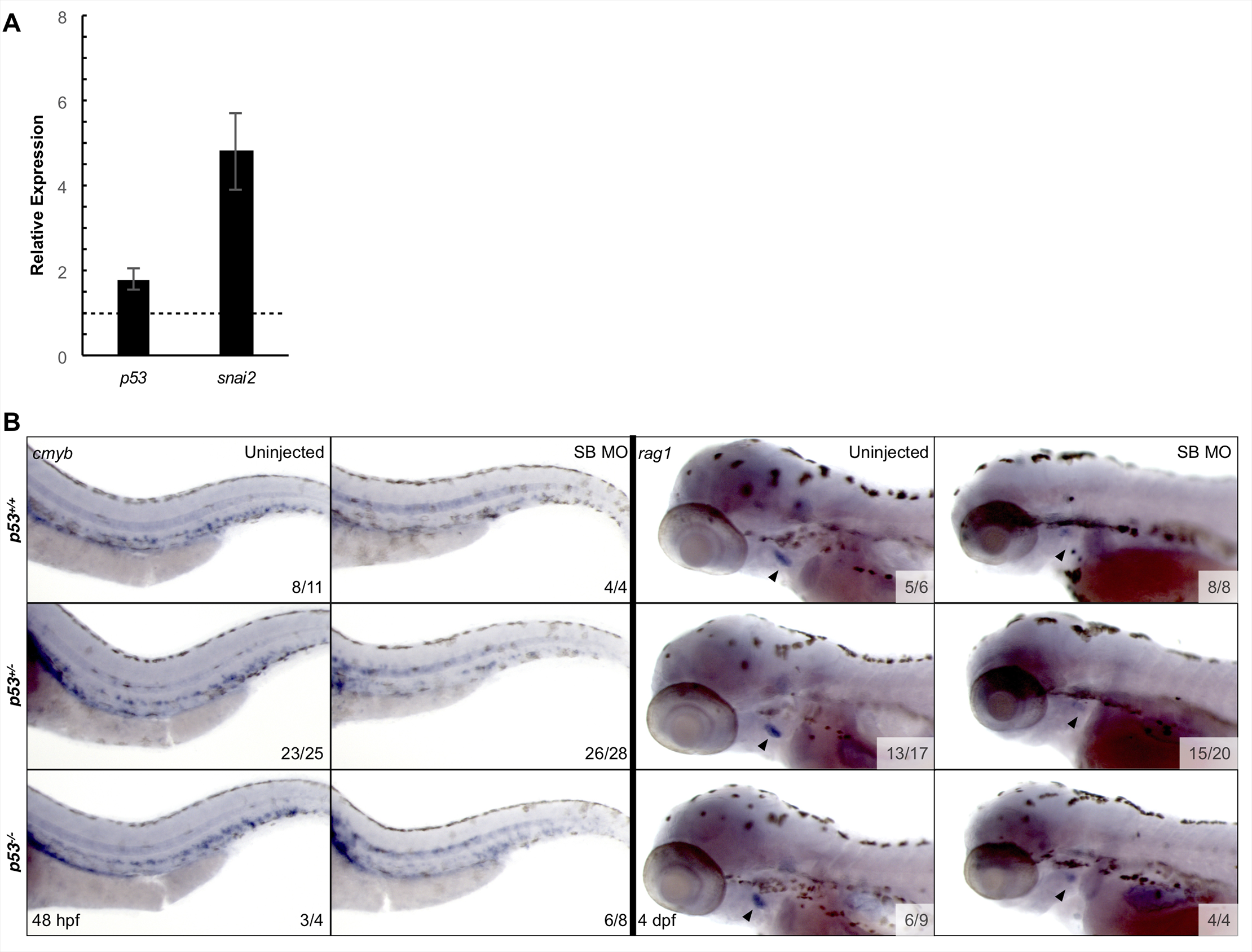

Supplement: S7 Fig — The potential of toxicity caused by the SB MO was analyzed by qPCR for p53 in morphant and uninjected pooled embryos at 26 hpf (A). Via this analysis, we saw that indeed p53 transcript was increased as compared to uninjected siblings. We also show snai2 transcript levels, as they are consistently increased in SB MO injected embryos. SB MO was then injected into embryos derived from a p53+/- in-cross, and the embryos analyzed for the hematopoietic markers rag1 and cmyb. The loss of p53 did not appear to rescue the morphant phenotype of decreased levels of both genes. Black arrowheads indicate the general position of the thymus. (TIF) [file pone.0202747.s007.tif]

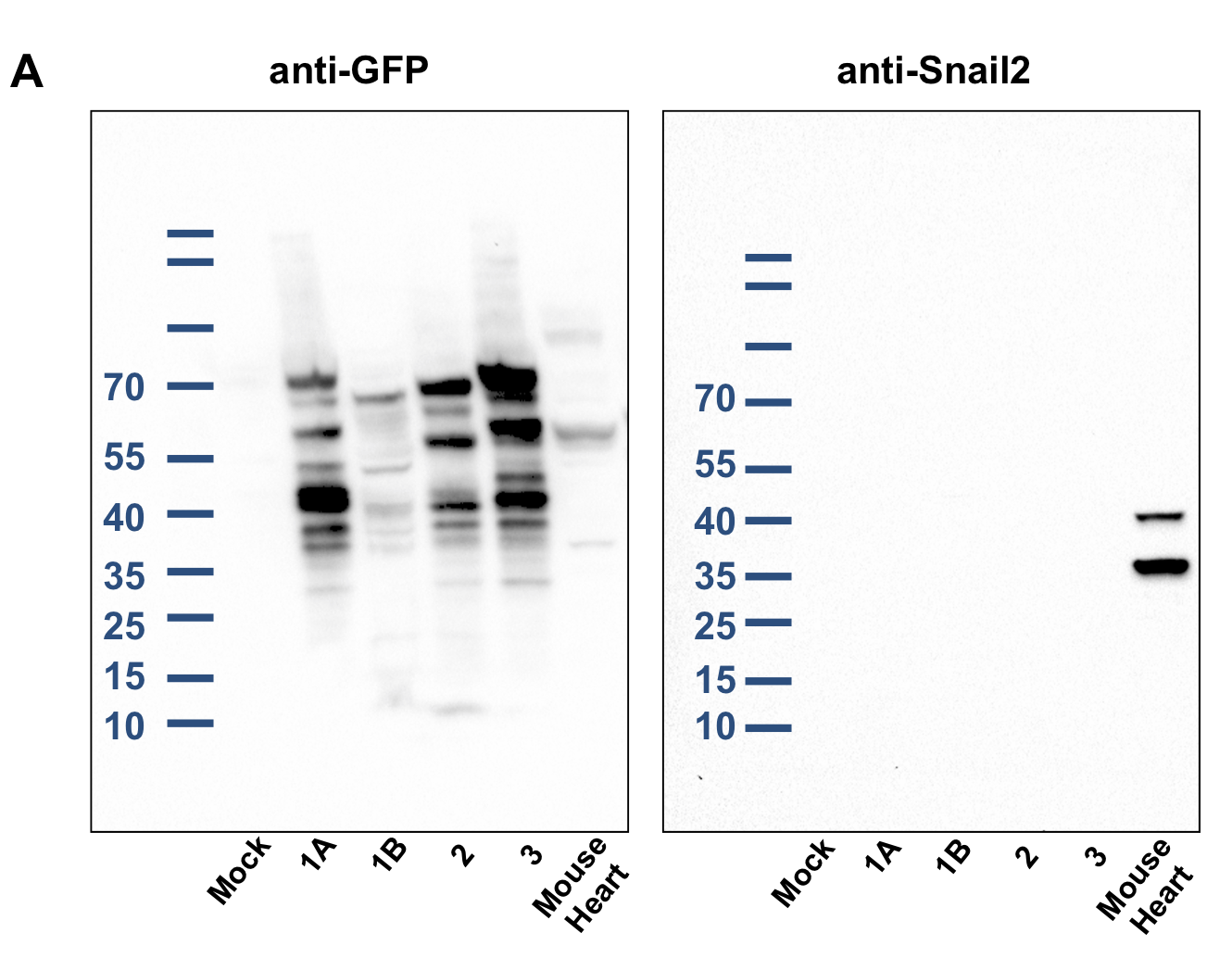

Supplement: S8 Fig — Protein lysates from HEK-293T cells transfected with the zebrafish Snail proteins N-terminally tagged with green fluorescent protein (GFP) were analyzed via western blot for both GFP and Snail2. Mouse heart lysate was also run as a positive control for Snail2 presence. Protein size is indicated to the left of each blot. Lanes are labeled with the appropriate number representing the zebrafish Snail family member. (TIFF) [file pone.0202747.s008.tiff]
